# Prevalence of Fleas and Gastrointestinal Parasites in Free-Roaming Cats in Central Mexico

**DOI:** 10.1371/journal.pone.0060744

**Published:** 2013-04-03

**Authors:** Germinal J. Cantó, Roberto I. Guerrero, Andrea M. Olvera-Ramírez, Feliciano Milián, Juan Mosqueda, Gabriela Aguilar-Tipacamú

**Affiliations:** Facultad de Ciencias Naturales, Universidad Autónoma de Querétaro, Cuerpo Académico Salud Animal y Microbiología Ambiental, Queretaro, Qro, México; Washington State University, United States of America

## Abstract

The prevalence of fleas and gastrointestinal parasites in free-roaming and domestic cats in central Mexico was evaluated. Three hundred and fifty eight cats captured in the street or brought in by owners to the Animal Control Center Unit, a unit of State Government, from June 2010 to May 2011, were included in the study. All cats were examined for the presence of fleas and gastrointestinal worms. One-hundred and ninety (53%) cats were infested with at least one flea species. Single infestations were observed in 106 (30%) cats and mixed infestations in 84 (23%) cats. Four species of fleas were recovered: *Ctenocephalides felis* in 53% of the cats, *C. canis* in 18%, *Echidnophaga gallinacea* in 7% and *Pulex irritans* in 1%. One-hundred and sixty three (45%) cats were infected with one or more species of gastrointestinal parasites: 48 (13%) with nematodes, 145 (40%) with cestodes, and one animal presented *Moniliformis moniliformis.* Prevalences and mean intensity of infection were: *Physaloptera praeputialis* 7 and 18; *T. cati* 3 and 2; *Ancylostoma tubaeforme* 2.5 and 2; *Toxascaris leonina* 0.5 and 2; *Dipylidium caninum* 36 and 32; *Taenia taeniformis* 4 and 3 and *Moniliformis moniliformis* 0.3 and 106, respectively. There was significant association (P<0.01), between season and ectoparasites load, more fleas were obtained in the summer and autumn than in the winter and spring; however, no statistical difference was observed for endoparasites load (P>0.05). The correlation between the total number of ectoparasites and endoparasites was not significant (r = 0.089, P = 0.094).

## Introduction

Companion animals, such as dogs and cats play an important role in society enhancing psychological and physiological welfare [1], [2]. However, pets play also a risk to public health [3] due to bites, scratches, allergies and infections to human beings [1], [3]. From the veterinary point of view, gastrointestinal parasites are the most common cause of disease in domestic cats. Stray cats are potential reservoirs for helminthic parasites, which can then be transmitted to domestic cats [4] and catś owners [5].

Free-roaming cats are either domestic cats that are not confined to an owner’s house or stray. They may be owned, but allowed to roam freely or they may be stray or recently owned, but lost or abandoned [6], [7]. Stray cats may include animals cared for and considered to belong to the neighborhood [7]. Free-roaming cats represent a reservoir of parasites potentially harmful to humans in developing countries, especially for children and the elderly, and also detrimental to other companion animals [5].

The prevalence of fleas and gastrointestinal parasites in free-roaming cats varies according to geographic location and season; therefore, each study may differ in the spectrum of species and prevalence affecting local cat populations [8], [9].

Information regarding prevalence and species of fleas and gastrointestinal parasites in cats in Mexico is scarce [10], [11], [12]; therefore, local and updated information is essential to understand the epidemiology of intestinal parasitic diseases in cats to design rational control strategies. This information is also important to prevent the indiscriminant use of anthelmintics that could lead to anthelmintic resistance [13], [14], [15]. Therefore, the objective of this study was to determine the species and prevalence of fleas and gastrointestinal parasites in free-roaming cats in the city of Queretaro, Mexico by origin, season, age and sex.

## Materials and Methods

Three-hundred and fifty eight free-roaming cats captured or brought in by owners to the Animal Control Center Unit (ACCU), a unit of the State Government, from June 2010 to May 2011, were included in the study: 273 were captured (strays cats) and 85 were brought in by the owners (domestic cats). Stray cats were captured in special cages by ACCU personnel and euthanized as part of a routine procedure. Domestic cats brought in by owners were euthanized with the owneŕs consent. One-hundred and fifty eight cats were male and 200 female. Age was determined as described by Eldredge [16]; 167 were between 1 and 7 months old (young), and 191 were adults. All cats were humanely euthanized; first, they were sedated with tiletamine-zolazepan and sacrificed with a lethal intracardiac injection of barbiturate, according to protocol NOM-033-200-1995 of the Mexican Ministry of Health [17]. To recover fleas, animals were combed craniocaudally with a plastic fine-toothed flea-comb for at least 15 minutes on the dorsal and ventral trunk. This was performed after sedation previous to sacrifice. Collected fleas were preserved in glass containers with a 70% ethanol solution until identification. Species identification of fleas was based on microscopic examination as described by Wall and Shearer [18]. Necropsy of all cats was performed according to Aluja [19]: trachea, lungs, heart and the complete digestive tract were separated and maintained at 4°C. The digestive tract was opened longitudinally and examined for the presence of helminths. Parasites found were preserved in a 70% ethanol solution until identification by microscopic examinations as described by Coffin [20]. Identification of *Moniliformis moliniformis* was based on size (10–12 cm), color (white) and the rosary-like appearance, armed proboscis and egg shape.

Infestation and infection level of ecto and endo parasites were determined according to origin (domestic or stray), age (young and adult), gender (male and female) and time of the year (four seasons). Prevalence was estimated by dividing the number of animals presenting an ecto or endoparasite by the total number of cats examined [21]. Abundance and mean intensity were estimated according to Bush [22]. Abundance was calculated by dividing the number of parasites of a particular species by the total number of cats examined. Mean intensity of infection was estimated by dividing the total number of parasites of a particular species by the number of cats infected with that parasite.

A Z-test was used to compare prevalence of ectoparasites and endoparasites load by origin, gender and sex using Epidat 3.1. A chi-square test was performed to determine the association ectoparasites and endoparasites loads and season. The correlation between the total number of ectoparasites and the total number of endoparasites was obtained in SPSS v16. The mean intensity of infection by origin, sex and age were compared throughout a student t-test.

## Results

### Ectoparasites

The overall prevalence of ectoparasite infestation was 53%. Fleas were the only ectoparasites found, 41 (48%) of the 85 domestic cats and 149 (55%) of the 273 stray cats, respectively were infested. No significant difference between groups was observed (P = 0.36). Single infestation was observed in 106 (30%) of the animals, while 84 (23%) harbored mixed infestations. Four species of fleas were recovered, *Ctenocephalides felis* was found in 189 (53%) of the cats, *C. canis* in 65 (18%), *Echidnophaga gallinacea* in 27 (7%) and *Pulex irritans* in 2 (1%) of the cats. A total of 2,985 fleas were recovered, *C. felis* was the most common flea observed with 2,670 (89%) specimens, followed by *C. canis* with 186 (6%) specimens, *E. gallinacea* with 127 (4%) and *Pulex irritans* with 2 (1%). No difference between prevalence of stray and domestic cats were found with unique infections (P = 0.14); however, a difference was observed in the case of mixed infestations (P = 0.0056).

Seasonal prevalence by gender, sex and age are shown in Table 1. Association between season and ectoparasites load was significant (P = 0.0001); however, no significant difference was observed for fleas load when comparing summer vs autumn (P = 0.98) and winter vs spring (P = 0.74). A significant difference was observed when the summer-autumn fleas load was compared to the winter-spring fleas load in a Z-test (P = 0.00001).

**Table 1:** Seasonal prevalence of flea infested cats in central Mexico by origin, sex and age.

	Domestic				Stray				
	Male		Female		Male		Female		
	Young (%)	Adult (%)	Young (%)	Adult (%)	Young (%)	Adult (%)	Young (%)	Adult (%)	Total %
SummerJun-Aug	0/0 (0.0)	4/8 (3.1)	3/5 (2.34)	9/18 (7.0)	14/28 (10.9)	18/20(14.1)	23/27 (18.0)	12/22 (9.4)	64.8^a^
AutumnSep- Nov	6/7 (11.3)	3/7(5.7)	1/4 (1.89)	10/14 (18.9)	2/3 (3.8)	4/5 (7.5)	5/6 (9.4)	4/7 (7.5)	66.0^ a^
WinterDec-Feb	0/0 (0.0)	1/5 (1.3)	0/0 (0.0)	1/4 (1.3)	6/13 (8.2)	6/12 (8.2)	5/16 (6.8)	15/26 (20.5)	46.3^ b^
SpringMar-May	0/2 (0.0)	2/3 (2.0)	1/4 (1.0)	0/4 (0.0)	13/28 (12.9)	8/17 (7.9)	9/24 (8.9)	5/19 (4.9)	37.6^ b^

ab Total season proportions with the same literal are not statistically different (P*>*0.05).

### Helminths

Three-hundred and fifty eight animals were examined for the presence of helminths, 163 (45%) were infected with one or more species, 48 (13%) presented nematodes and 145 (40%) cestodes. *D. caninum, P. praeputialis* and *T. taeniformis* were the most common cestods, with prevalences of 36%, 7.0% and 4% respectively. Prevalence for the rest of the parasites, in descending order were *T. cati* 3%, *A. tubaeforme* 2%, *T. leonina* 1% and *M. moniliformis* 0.3% (Table 2). No parasites were found in the respiratory tract and heart.

**Table 2:** Prevalence of unique and mixed helminth infection recovered in domestic and stray cats in central Mexico.

Helminth infection	Domestic, 85 (24%)		Stray, 273 (76%)	
	Infested cats	Prevalence %	Infested cats	Prevalence%
	**Unique infections**			
*Toxocara cati*	0	0	6	2.2
*Toxascaris leonine*	0	0	1	0.4
*Taenia taeniformis*	1	1.2	8	2.9
*Physaloptera praeputialis*	4	4.7	9	3.3
*Dipylidium caninum*	25	29.4	79	29
*Ancylostoma tubaeforme*	2	2.4	3	1.1
*Moniliformis moniliformis*	0	0	0	0
**Total unique infection**	**32**		**116**	
	**Mixed infections**			
*Dipylidium caninum-Taenia taeniformis*	0	0	6	2.2
*Dipylidium caninum-Toxocara cati*	0	0	4	1.5
*Dipylidium caninum-Physaloptera praeputialis*	3	3.5	8	3
*Dipylidium caninum_Ancylostoma tubaeforme*	0	0	3	1.1
*Dipylidium caninum-Toxocara cati-Toxascaris leonina*	1	1.2	0	0
*Dipylidium caninum-Physaloptera praeputialis-Moniliformis moniliformis*	0	0	1	0.4
*Ancylostoma caninum-Toxocara cati*	1	1.2	0	0
**Total mixed infections**	**5**		**22**	

Single infections were observed in 129 (36%) and mixed infections in 27 (7%) of the cats. Statistical differences between the proportion of infected stray and domestic cats was only observed in mixed infections (P =  0.000); the largest number of mixed infections was observed in stray cats.

Seasonal prevalence by gender, sex and age are shown in Table 3. The association between season of the year and endoparasite load was not significant (P = 0.055). No statistical differences was observed between season and endo parasites load by gender (P = 0.76), sex (P = 0.14) and age (P = 0.14).

**Table 3:** Seasonal prevalence of gastrointestinal parasites infested cats in central Mexico by origin, sex and age.

	Domestic				Stray				
	Male		Female		Male		Female		
	Young	Adult	Young	Adult	Young	Adult	Young	Adult	Total %
SummerJun-Aug	0/0 (0.0)	4/8 (3.1)	2/5 (1.6)	9/18 (7.0)	9/28 (7.0)	10/20(7.8)	11/27 (8.6)	11/22 (8.6)	43.8
AutumnSep- Nov	6/7 (11.4)	3/7(5.7)	1/4 (1.9)	9/14 (17)	1/3 (1.9)	5/5 (9.4)	3/6 (5.7)	5/7 (9.4)	62.3
WinterDec-Feb	0/0 (0.0)	0/5 (0.0)	0/0 (0.0)	1/4 (1.3)	5/13 (6.6)	7/12 (9.2)	5/16 (6.6)	16/26 (21.1)	44.7
SpringMar-May	0/2 (0.0)	1/3 (1.0)	0/4 (0.0)	1/4 (1.0)	14/28 (13.9)	17/17 (16.8)	7/24 (7.0)	10/19 (10.0)	40

The mean of parasite intensity and abundance by species are shown in Table 4. A total of 4,782 worms were recovered. *M. moniliformis* presented the highest intensity of infection with 106 specimens, followed by *D.caninum* with 32, *P. praeputialis* with 18, *T. taeniformis* with 3 and *A. tubaeforme* with 2 parasites. *T. canis* and *T. leonina*, presented a mean intensity of 2. The abundance of infection was higher for *D. caninum* and *P. praeputialis,* 11.5 and 1.3 parasites, respectively. The rest of the helminths had an abundance of infection below 0.3. The mean intensity of infection by origin, sex and age is presented in Table 5. Results of the *t-*tests showed statistical difference only when adult stray males and females were compared for *D. caninum* (P = 0.027) and *P. praeputialis* (P = 0.038). No correlation was observed between the total number of ectoparasites and the total number of endoparasites in the total group of cats (r = 0.089, P = 0.094).

**Table 4:** Mean intensity and abundance of gastrointestinal worms in free-roaming cats in central Mexico.

Parasite species	Mean intensity(+ SD)	Abundance(+ SD)
*Physaloptera praeputialis* (25)*	18.44 (32.21)	1.28 (9.58)
*Toxocara cati*(12)	2.00 (1.59)	0.07 (0.46)
*Toxascaris leonina* (2)	2.00 (1.41)	0.01 (0.17)
*Ancylostoma tubaeforme* (9)	2.44 (2.24)	0.06 (0.51)
*Dipyliduim caninum* (130)	31.68 (37.89)	11.50 (27.42)
*Taenia taeniformis* (15)	3.06 (2.09)	0.13 (0.74)
*Moniliformis moniliformis*(1)	106	0.30 (5.60)

* Number of cats with that specific parasite

**Table 5:** Mean intensity of gastrointestinal worms in free-roaming cats in central Mexico by origin, sex and age.

Parasite species	Domestic				Stray			
	Male		Female		Male		Female	
	Young	Adult	Young	Adult	Young	Adult	Young	Adult
*Ancylostoma tubaeforme*	0	0	0	1	3.5	4	1	6
*Toxocara cati*	0	1	0	2	1.8	1	6	2
*Toxascaris leonine*	0	3	0	0	0	1	0	0
*Physaloptera praeputialis*	0	1	0	47.2	1	4	2.7	18.4
*Dipylidium caninum*	21.3	26.3	15.3	30.6	19.3	25.8	34.7	49.6
*Taenia taeniformis*	0	0	0	1	3	2	5	3.6
*Moniliformis moniliformis*	0	0	0	0	0	0	106	0

## Discussion

Most studies about ectoparasites or flea infestations in cats have shown variable frequencies, *C. felis* has been found by far to be the most prevalent flea species [23], [24], [25], [26], [27], [28]. In Mexico, Cruz-Vazquez [10] reported a prevalence of 30.3% of ectoparasites in a three years study in Cuernavaca, Morelos, Mexico, where 92% of the cats were infested with *C. felis* and 8% with *C. canis*, in our study, the flea prevalence was considerably higher (53%). *C. felis* was found in 99% of the infested cats, in agreement with previous reports [26], [29], [30], [31], [32]. *C. canis* was found in 34% and *E. gallinacea* in 14% of the cats in our study. Previous studies have shown that *C. felis* is the most common flea in urban areas whereas *C. canis* is the most common in rural areas [33], [34], [35]. However, there are studies that did not find differences in infestation rates between urban and rural areas [24], [36]. Even though we did not study cats from rural areas, it is an issue that *C. felis* is the most important flea in Mexico and represents a public health concern because it has been reported as the principal host of *Rickettsia felis*, which causes rickettsiosis in humans in the south of Mexico [37], [38]. The third most common flea, *E. gallinacea*, the stick tight flea, is a cosmopolitan flea, primarily a pest of domestic poultry and birds [39], [40] but may also parasitize mammals [41] such as dogs [42], [43], fox and rats [41]. Studies in Europe and America were not able to find this flea in flea infested cats [30] [10], [31]. However, Akucewich [23] have reported *E. gallinacea* (5.5%) in cats in the north central of Florida, in the United States, suggesting that the presence of the flea in feral cats is caused for the close contact with wild and domestic birds. This is the first time that *E. gallinacea* is reported infesting cats in Mexico.

Statistical differences of flea infestations between seasons were found, the highest proportion of infested cats was observed in the summer and autumn. Xaxhiou [27] found major prevalence of flea infestation during summer in Albania. Akucewich [23] showed major numbers of *C. felis* between June and July, in The United States. Temperatures ranging from 20 to 30°C [44] and relative humidity in excess of 50% are required for optimum larval development of these fleas [45]. In Queretaro, the relative humidity and the mean temperature in the summer are 65% and 19°C, respectively [46]. These parameters in autumn are 62% and 16°C, in the winter 45 and 13°C, and in the spring 42% and 20°C respectively [46]. Silverman and Rust [47] found that 70% of the flea eggs hatch at 16°C and 33% relative humidity. They also found that the eggs survival was up to 92% when the relative humidity is higher. However, with 27°C and 50% relative humidity the majority of the eggs hatch.

Parasite identification post-mortem has advantages over fecal examination. In post-mortem identification all parasites can be observed and are not inferred from egg observation. It has been reported that fecal examination has poor sensitivity [48], [49] in estimating the number of parasites in an infected animal.

Forty five percent of the euthanized cats presented at least one species of gastrointestinal parasite; these results are similar to those observed by Nichol [50]; however they are higher than those found by others [51], [52], [53]; nevertheless, some authors have reported even higher proportions [4], [8], [9]. The main gastrointestinal parasites found in this study were cestodes; however, our prevalence was lower than that reported by Abu-Madi [9] (97%), who suggested that the hostile nature of the external environment, which impact survival of directly transmitted helminthes, is responsible for that high prevalence.

The proportion of single (36%) and multiple (7%) infections differed greatly. These results may indicate that cats have a high level of resistance against mixed infections as suggested by Engbaek [54]. However, our results differ from those reported by Abu-Madi [9] in the city of Doha, Qatar, where 37% of the cats showed two parasite species.

Differences in infection intensity between males and females with *D. caninum* and *P. praeputialis*, in stray cats where observed. This could be due to the fact that females are more social and less mobile when they have kittens building up the flea population in the nest [54].

*D. caninum,* and *P. praeputialis* , were the two most common endo parasites found in this study. This could reflect the ingestion of the intermediate hosts, fleas and lice in the case of *D. caninum* and beetles and cockroaches in the case of *P. praeputiales* [8]. Rodríguez-Vivas [12] found *Ancylostoma* spp. (33%), *Toxascaris leonina* (22%) and *D. caninum* (17%) in cats in Yucatán, Mexico. The most common endoparasites reported in Venezuela were, *Ancylostoma* (30%) and *Trichuris* (11%) [55] and in England, *D. caninum* (35%) was the most common endoparasite in feral cats [50]. In addition, *D. caninum* is frequently found in mixed infections [8] which could be related to a large flea population. In our study *D. caninum* was found in mixed infections, mainly with *Taenia taeniformis*, *Toxocara cati* and *Physaloptera praeputialis.*

*P. praeputialis* was the parasite with the second highest prevalence, intensity of infection and abundance. There are few reports of this parasite in Mexico [11], [56]. It has been suggested that this parasite is frequently underestimated due to the small size and lack of color of the eggs, which are overlooked in fecal analysis [8].

*Toxocara cati,* often reported as one of the most frequently found parasite of cats [53], [57], [58]was found in only 12 cats in our study; however, this is one of the most important parasite in public health, especially in free-roaming cats with free access to public parks where young children congregate [59]. *Moniliformis moniliformis* was found in the small intestine of one cat with 106 more parasites. *M. moniliformis* may affect humans, causing abdominal pain, diarrhea and vomiting [2]. To the best of our knowledge, this is the first time that *M. moniliformis* has been reported in Mexico.

Results of this study show that *C. felis is* the most frequent ectoparasite and *D. caninum* the most frequent endoparasite in cats in Queretaro, Mexico. Nevertheless, no correlation was found between ectoparasite and endoparasite burden. In conclusion, our results provide important information about the prevalence and the kind of parasites present in free roaming cats in central Mexico, and provide the basis for additional work focus in developing control programs to prevent risks to public health.
